# The Role of Volunteers in Supporting the Psychosocial Well-Being of Hospitalised Children with Cancer: A Narrative Review and Recommendations for Clinical Practice

**DOI:** 10.3390/children13070928

**Published:** 2026-07-15

**Authors:** Ivana Kreft Hausmeister, Janez Jazbec

**Affiliations:** Clinical Department of Paediatric Haematology and Oncology, Paediatric Clinic, University Medical Centre Ljubljana, Zaloška 2, 1000 Ljubljana, Slovenia; ivana.kreft@kclj.si

**Keywords:** paediatric oncology, hospitalised children, volunteers, psychosocial support, cancer nursing, play interventions, distraction, clinical recommendations

## Abstract

Children undergoing cancer treatment face repeated, prolonged hospitalisations carrying substantial risks of psychological distress, social isolation, and impaired development. Volunteers offering structured non-medical activities are widely employed to complement professional psychosocial care in paediatric oncology settings, yet empirical evidence examining their role specifically—as distinct from professionally delivered psychosocial interventions—remains sparse. This narrative review, conducted in accordance with SANRA criteria and drawing on a structured search of PubMed, PsycINFO, and Google Scholar (2000–2025), supplemented by a targeted verification search in July 2026, synthesises the theoretical and preliminary empirical basis for volunteer involvement, critically evaluates associated clinical risks, and proposes evidence-informed recommendations for paediatric oncology clinical practice. Three theoretical frameworks provide the conceptual grounding for volunteer activities: developmental psychological models (Piaget; Erikson), stress and coping theory (Lazarus and Folkman), and the biopsychosocial model (Engel). Indirect empirical evidence—derived predominantly from professionally delivered interventions—supports short-term benefits of structured distraction, play, and social activities on procedural anxiety, pain experience, and sense of social inclusion; the sole study examining volunteer-facilitated activities specifically in paediatric oncology reported meaningful reductions in distress and high family satisfaction. Eight categories of clinical risk are identified, including physical and psychological overload, erosion of child autonomy, suppression of authentic emotional expression, and inadequate coordination with healthcare staff. Six practice recommendations are proposed, centred on a working definition of the volunteer role, mandatory pre-access training, genuine individualisation including cultural adaptation, multidisciplinary-team-led coordination, and structured programme evaluation. Volunteer involvement can meaningfully complement professional psychosocial care when delivered within a structured, supervised, and multidisciplinary-team-coordinated framework. The paediatric oncology team occupies a pivotal role in realising the potential benefits while safeguarding children from associated risks. Prospective research employing standardised volunteer programme definitions and validated child-reported outcome measures is urgently needed to build an evidence base commensurate with the clinical importance of this practice.

## 1. Introduction

Advances in the treatment of paediatric malignancies have substantially improved survival rates over recent decades, with five-year survival now exceeding 80% in high-income countries [1]. This oncological achievement has introduced a parallel imperative: ensuring the highest possible quality of life for children during treatment. Hospitalisation often involves prolonged separation from home, family, and peers; repeated invasive diagnostic and therapeutic procedures; distressing medication side effects; and sustained uncertainty regarding prognosis [2]. These experiences constitute significant psychosocial stressors, with documented effects on anxiety, depression, post-traumatic stress, and long-term psychological adjustment [2,3].

In response, comprehensive paediatric oncology care increasingly incorporates psychosocial elements alongside biomedical treatment. Psychologists, social workers, teachers, and play therapists provide professional psychosocial support; however, healthcare systems also rely on volunteers to extend the range and availability of non-medical activities. Play, music, art, storytelling, and peer interaction are offered through both professional and volunteer pathways, and may collectively support the child’s psychological adjustment and sense of normality during hospitalisation [4,5].

Despite growing interest in volunteer involvement in hospital settings, a critical gap exists in the evidence base: studies examining volunteer-delivered activities specifically—as distinct from professionally delivered psychosocial interventions—in paediatric oncology are rare, and the field lacks both standardised programme definitions and validated volunteer-specific outcome measures. The majority of available evidence examines activities that volunteers are commonly asked to deliver, but under professional rather than volunteer direction; this indirect evidence base provides theoretical and empirical grounding for volunteer involvement while stopping short of demonstrating its effects directly. Furthermore, the conditions under which volunteer involvement is beneficial, safe, and appropriately coordinated with professional care remain poorly defined. This review therefore approaches the evidence base transparently: drawing on indirect empirical evidence where direct evidence is absent, making that distinction explicit throughout, and grounding clinical recommendations in theoretical frameworks and risk analysis where the empirical literature does not yet reach. This is particularly relevant for the paediatric oncology team, who bear central responsibility for coordinating the child’s care environment and are frequently the clinical professionals most proximate to volunteer interactions on the ward.

The aim of this narrative review is to examine the theoretical and empirical basis for volunteer involvement in the psychosocial support of hospitalised children with cancer; to critically evaluate associated clinical risks; and to propose evidence-informed, practice-ready recommendations for paediatric oncology clinical practice. We argue that the multidisciplinary paediatric oncology team is uniquely positioned to lead the implementation of structured, safe, and individualised volunteer programmes.

## 2. Methods

This narrative review was conducted in accordance with established methodological guidance for narrative reviews in health sciences [6]. For the purposes of this review, a volunteer is defined as an unpaid individual providing structured non-medical activities to hospitalised children in a lay capacity—that is, without the clinical training of a psychologist, music therapist, or play specialist, and outside any formal educational placement. Parent volunteers and medical students on clinical placement are therefore excluded from this definition. This operational definition was applied consistently in screening and interpreting the evidence.

A narrative rather than systematic review design was selected for three reasons: the primary objective was theoretical synthesis and the development of practice recommendations rather than effect-size estimation; the available evidence is insufficiently homogeneous in design, population, and outcome measurement to support meta-analytic pooling; and narrative review is the methodologically appropriate form for fields at an early stage of evidence development, where mapping the conceptual and empirical landscape serves clinical utility better than premature quantitative synthesis [6].

A structured literature search was performed across PubMed, PsycINFO, and Google Scholar in February and March 2025, using combinations of the following terms: volunteers, paediatric/pediatric oncology, hospitalised/hospitalized children, psychosocial support, distraction, play interventions, cancer nursing, anxiety, pain, well-being, psychological adjustment. Foundational theoretical texts predating 2000 were included where directly relevant to the conceptual framework. Google Scholar was included alongside PubMed and PsycINFO to reduce the risk of missing relevant grey literature and non-indexed sources; we acknowledge, however, that its proprietary and non-transparent ranking algorithm means search results are not fully reproducible in the way that PubMed and PsycINFO searches are, a limitation inherent to narrative reviews that draw on this database and one we flag explicitly here rather than presenting the search as equivalent in rigour across all three sources. Articles were screened for relevance to the role of volunteers or structured non-medical activities on the psychosocial well-being of children with cancer. Case reports and editorials were excluded. A key finding of the search was that empirical studies examining volunteer-delivered interventions specifically are rare; the majority of identified evidence examines professionally delivered psychosocial activities. This evidence base is included because it provides the best available empirical grounding for the activities volunteers are asked to deliver, but findings are interpreted throughout with explicit acknowledgement of this distinction. Thematic categories were developed inductively from the evidence to address the review’s objectives.

To improve methodological transparency in accordance with SANRA recommendations, the following details of the selection process are reported. Consistent with the narrative, non-systematic design justified above, the broader evidence base synthesised in this review (Table 1) was assembled through iterative searching across combinations of the listed terms together with citation-chasing from the reference lists of key papers, rather than through a single reproducible Boolean query; exact retrieval and exclusion counts for this exploratory process were not tracked at the time and cannot be reconstructed retrospectively. As a targeted supplementary check, a reproducible verification query combining the volunteer concept with the population terms (volunteer* AND paediatric/pediatric oncology terms AND hospitalised/hospitalized terms) was run in PubMed on 10 July 2026 and returned only 2 records, neither of which examined volunteer-delivered activity as operationalised in this review; this small, targeted result is consistent with, and offers concrete quantitative support for, the review’s central finding that volunteer-specific empirical evidence is sparse. Study selection was performed independently by both authors (I.K.H. and J.J.), with disagreements resolved by discussion and consensus. Database searching and study selection were conducted manually by the authors; a generative AI tool was used only as a supplementary check for potentially missed records after the authors’ own search was complete, and was not used as a systematic tool for literature identification or selection (see Declaration of Generative AI and AI-Assisted Technologies below).

The conduct and reporting of this narrative review followed the SANRA (Scale for the Assessment of Narrative Review Articles) criteria, which provide a structured framework for the transparent reporting of non-systematic literature reviews [20]. The selection of included sources, the rationale for inclusion and exclusion decisions, and the characteristics of each reviewed study are reported in Table 1, in accordance with the principle of methodological transparency recommended for narrative reviews in health sciences. Theoretical foundational works (monographs by Piaget, Erikson, Lazarus and Folkman, and Engel) were included on the basis of their direct relevance to the conceptual framework rather than through the database search, and are noted as such in Table 1.

## 3. Theoretical Framework

Understanding the mechanisms through which volunteer activities may benefit children with cancer requires integration of several theoretical perspectives. These three frameworks were selected, rather than other developmental or psychosocial models, because each addresses a distinct and complementary mechanism directly relevant to volunteer-delivered activity: developmental theory explains why play and social interaction matter to the child as a developing person; stress and coping theory explains how specific volunteer activities such as distraction and music act on the child’s momentary experience of a stressor; and the biopsychosocial model situates volunteer involvement within the broader interaction of psychological, social, and physical dimensions of illness that is central to paediatric oncology care. Together they span the individual, the encounter, and the illness context, providing a more complete theoretical grounding than any single perspective alone.

### 3.1. Developmental Psychological Models

Piaget and Erikson’s foundational models of child development emphasise that play and social interaction are not incidental to childhood, but fundamental mechanisms through which children process experience, develop cognitive schemas, regulate emotion, and maintain identity [16,17]. Hospitalisation disrupts these developmental processes by restricting the child’s agency, social world, and opportunity for normative peer interaction. Volunteer activities that restore opportunities for play, choice, and social engagement may therefore buffer the developmental impact of prolonged hospitalisation. Critically, the opportunity to *choose* to participate—and equally to decline—is theoretically central to the restoration of a sense of control and autonomy.

### 3.2. Stress and Coping Theory

Lazarus and Folkman’s transactional model of stress and coping posits that psychological distress is determined not by objective stressors alone, but by the child’s cognitive appraisal of those stressors and by available coping resources [18]. Distraction-based volunteer activities—play, music, storytelling—function as emotion-focused coping strategies, reducing the subjective salience of painful or threatening stimuli. Evidence supports the effectiveness of distraction in reducing procedural pain and anticipatory anxiety, particularly when initiated before rather than during the aversive event [8,9]. Social support from volunteers may additionally enhance the child’s sense of being cared for and understood, supporting more positive reappraisal of the illness experience.

### 3.3. The Biopsychosocial Model

Engel’s biopsychosocial model highlights that physical, psychological, and social dimensions of illness are interdependent [19]. In paediatric oncology, this interdependence is particularly salient: psychological distress directly influences physiological parameters including pain sensitivity, immune function, and treatment compliance, while physical symptoms profoundly affect psychological state. Volunteer involvement, when appropriately structured, addresses the social dimension of the biopsychosocial triad—reducing isolation, fostering connection, and supporting positive affect—with potential downstream effects on physical well-being. It is within this integrated model that volunteer involvement finds its strongest theoretical justification as a complement to biomedical treatment.

## 4. Evidence for the Psychosocial Benefits of Volunteer Activities

### 4.1. Anxiety and Stress Reduction

The strongest available evidence for anxiety reduction through structured play and distraction derives from studies of professionally delivered interventions. Li et al.’s quasi-experimental study of play interventions in hospitalised children found statistically significant reductions in anxiety and negative affect across a range of activity formats, with effects maintained across age subgroups [10]. It should be noted that Li et al.’s sample comprised mixed-diagnosis hospitalised children and did not distinguish between volunteer and professional delivery; direct extrapolation to volunteer-led programmes requires caution. The sole study in this review examining volunteer-facilitated activities specifically in paediatric oncology is Perasso et al., who reported meaningful reductions in distress and high family satisfaction in an Italian multidisciplinary programme involving trained oncology volunteers [5]. That study does not report a sample size, which limits the weight that can be placed on its findings. Taken together, the available evidence provides a theoretical and preliminary empirical rationale for volunteer-delivered distraction activities, while underscoring the need for volunteer-specific research. Benefits appear most pronounced when activities are offered proactively before invasive procedures rather than reactively during them.

### 4.2. Pain Experience and Procedural Tolerance

Distraction is among the best-evidenced non-pharmacological strategies for paediatric procedural pain, though the evidence base again derives predominantly from professionally delivered interventions. Uman et al.’s Cochrane review found high-quality evidence that distraction reduces needle-related pain and distress across diverse child ages and procedural types [8]. Nguyen et al.’s randomised trial demonstrated that music—an activity frequently facilitated by trained volunteers—significantly reduced pain scores and physiological arousal during lumbar puncture in children with leukaemia [9]. Neither study was designed to test volunteer delivery specifically; the evidence supports the activities themselves as effective, and by extension provides a rationale for volunteers trained to deliver them, but does not constitute direct evidence for volunteer programmes. These effects carry particular clinical significance in paediatric oncology, where repeated invasive procedures accumulate substantial procedural distress across the course of treatment.

### 4.3. Social Inclusion and Developmental Continuity

Prolonged hospitalisation imposes social isolation with consequences extending beyond acute distress: peer relationships deteriorate, social competencies may regress, and the child’s sense of belonging to a wider community erodes [11]. Coyne’s qualitative study of hospitalised children’s own perspectives found that peer interaction, play, and opportunities for normality were among the experiences children most valued and most missed during admission [11]. Volunteer activities—particularly those involving peer volunteers, hospital school programmes, or art and music workshops with external participants—are theoretically well-positioned to address precisely these deficits, by introducing social contact and normalising experiences that clinical staff and parents alone cannot fully provide. Phipps et al. found that the quality of psychosocial support during hospitalisation predicted long-term psychological adjustment in childhood cancer survivors, underscoring that social support during treatment has consequences beyond the admission itself [3]. Direct evidence that volunteer-delivered social activities specifically produce these outcomes is not yet available; the case rests on the convergence of children’s expressed needs, theoretical models, and the broader psychosocial support literature.

### 4.4. Summary of the Evidence Base

As noted throughout Section 4.1, Section 4.2 and Section 4.3, the indirect empirical evidence reviewed here derives predominantly from professionally delivered interventions rather than volunteer-specific programmes. Across 25 years of searching, only a small number of studies bear directly on volunteer-delivered activity, or activities volunteers are commonly asked to deliver, in paediatric oncology specifically (summarised in Table 1); we regard this as reflecting a genuine scarcity in the field rather than an artefact of search strategy, and the recommendations in Section 7 should be weighed accordingly. The sole study directly examining volunteer-facilitated activities in paediatric oncology is Perasso et al., whose findings are promising but limited by an unreported sample size [5]. The broader evidence base is additionally characterised by quasi-experimental designs, small and selectively recruited samples, short follow-up periods, and heterogeneous outcome measures [4]. The operational definition of volunteer activity varies substantially across studies, encompassing play, music, educational support, art workshops, and animal-assisted interactions, making aggregation difficult.

These limitations are not incidental to this review—they are its central finding. The field does not yet have the volunteer-specific evidence base that clinical practice in this area requires. The theoretical frameworks, indirect empirical evidence, and risk analysis presented in this review are offered as the most rigorous currently available grounding for practice recommendations, while the need for prospective volunteer-specific research with standardised programme definitions and validated child-reported outcome measures remains urgent.

### 4.5. Differential Considerations Across Diagnostic and Treatment Contexts

The evidence and risk analysis presented above has, to this point, treated hospitalised children with cancer as a relatively homogeneous population. In clinical practice, the feasibility and appropriate form of volunteer involvement differ substantially by diagnostic and treatment context, and this heterogeneity deserves explicit acknowledgement even though the evidence base does not yet allow a stratified analysis. Children undergoing treatment for haematological malignancies, particularly during and after haematopoietic stem cell transplantation or periods of profound post-chemotherapy neutropenia, are frequently subject to prolonged protective isolation under strict sanitary regimes that preclude direct physical contact with volunteers for extended periods. In this context, volunteer involvement may need to be reconceived around remote or window-mediated interventions—supervised video calls, remotely delivered storytelling or music, or recorded content relayed through nursing staff—rather than bedside activities. Children with solid tumours more often retain physical access to volunteers but may face distinct psychosocial challenges related to visible changes in body image, for example following surgery, amputation, or alopecia, or to neurological deficits arising from central nervous system tumours or their treatment; volunteers working with these children may require different communication and engagement training than those working primarily on a haematology–oncology ward. We did not identify empirical studies directly comparing volunteer programme design, delivery, or outcomes across these contexts. This is both a limitation of the current evidence base and a specific, concrete direction for future research and local programme design, and we flag it here as a direction rather than a finding of this review.

## 5. Critical Risks and Challenges in Involving Volunteers

Despite their potential benefits, volunteer programmes in paediatric oncology carry distinct risks that must be systematically addressed. We identify eight clinically significant risk categories.

### 5.1. Physical and Psychological Overload

Children undergoing intensive chemotherapy, radiotherapy, or haematopoietic stem cell transplantation are frequently in states of profound physical and cognitive depletion. Multiple consecutive or lengthy volunteer activities impose additional demands on children who may lack the resources to decline without feeling obligated. Excessive stimulation has been associated with increased fatigue, irritability, and reduced cooperation during subsequent medical procedures [14]. The clinical care team, who are best positioned to assess the child’s current functional reserve, must hold clear authority to suspend or redirect volunteer activities when clinical status warrants.

### 5.2. Erosion of Child Autonomy

Paradoxically, activities intended to restore agency may undermine it if participation is implicitly expected. Children who are unable or unwilling to engage—whether due to fatigue, low mood, or simple preference—may experience social pressure to participate that compounds, rather than alleviates, distress [11]. The genuinely voluntary character of every activity must be preserved, with active, age-appropriate consent obtained and respected unconditionally at each encounter.

### 5.3. Suppression of Authentic Emotional Expression

Volunteer interactions that emphasise positivity, entertainment, and distraction may inadvertently communicate to the child that expressions of fear, sadness, or anger are unwelcome. Bluebond-Langner et al. found that children with serious illness are acutely sensitive to the emotional expectations of those around them, and may suppress authentic emotional communication in order to protect caregivers’ wellbeing [15]. Volunteer training must explicitly address the importance of emotional openness and must not frame the volunteer’s role as ‘keeping the child happy’.

### 5.4. Inadequate Preparation and Training

Volunteers with optimal intentions but insufficient preparation may fail to recognise signs of distress, fatigue, or clinical deterioration; may inadvertently disclose clinical information; or may initiate interactions poorly matched to the child’s developmental stage or current condition. Mandatory structured training before ward access, covering recognition of distress signals, communication strategies, developmental considerations, and boundaries of the volunteer role, is a non-negotiable prerequisite [5].

### 5.5. Absence of Coordination and Institutional Structure

Volunteer presence without clear institutional guidelines, scheduling coordination, and regular communication with the ward team risks disrupting clinical workflows, duplicating or conflicting with professional psychosocial interventions, and generating confusion regarding role boundaries [5]. A designated volunteer coordinator role, ideally held by a senior nurse or clinical psychologist, provides the structural foundation for effective integration.

### 5.6. Insufficient Individualisation

Children’s developmental stages, temperaments, cultural backgrounds, disease phases, and personal histories vary substantially. An activity appropriate for a seven-year-old in remission may be wholly unsuitable for a fifteen-year-old immediately following a difficult procedure. Individual assessment, including input from the child and family, must precede and continuously inform volunteer activity planning [10].

### 5.7. Infection Control Risks

Children undergoing immunosuppressive treatment are at elevated risk from infectious agents. Volunteers with active respiratory infections, herpetic lesions, or recent exposure to communicable disease must not have contact with immunocompromised patients. This risk is most acute during periods of profound and prolonged neutropenia, such as following intensive chemotherapy or haematopoietic stem cell transplantation, when contact restrictions are typically most rigorous and may temporarily preclude any volunteer contact regardless of the volunteer’s own health status; protocols should specify objective clinical criteria (e.g., absolute neutrophil count thresholds, isolation-room status) that trigger automatic suspension of in-person volunteer contact, rather than leaving this judgement to case-by-case discretion. Institutional infection control policies must explicitly cover volunteer access, with consistent implementation and nurse oversight.

### 5.8. Conflict with Professional Healthcare Work

Poorly coordinated volunteer presence may disrupt routine clinical procedures, create role confusion among staff, or require additional staff time for management and redirection. Clear communication protocols, agreed scheduling, and explicit role boundaries—combined with regular joint team meetings between volunteer coordinators and the ward clinical team—are essential to prevent conflict and preserve clinical effectiveness [5].

## 6. The Central Role of the Multidisciplinary Paediatric Oncology Team

Nurses and other members of the clinical care team are the healthcare professionals most continuously present in the child’s care environment. This proximity positions the clinical team as the natural lead in the coordination, oversight, and evaluation of volunteer involvement, with nurses typically holding the most sustained day-to-day contact. Across four dimensions, multidisciplinary engagement is essential.

**Assessment and gatekeeping.** The clinical care team, and nurses in particular, are best placed to assess the child’s daily clinical and psychological state, and to determine whether volunteer contact is appropriate, beneficial, or should be withheld on a given day. This clinical authority must be clearly recognised and respected within volunteer programme structures.

**Coordination and communication.** The clinical team, often led by nursing staff, serves as the communication bridge between the volunteer team, the broader multidisciplinary team, and the family. Clear handover of relevant clinical information—within the bounds of confidentiality—enables volunteers to adapt their approach and timing appropriately.

**Supervision and debriefing.** Regular structured supervision of volunteers, including feedback on individual child responses and programme-level quality review, is a nursing and clinical psychology responsibility. Without supervision, volunteers may inadvertently perpetuate approaches that are mismatched to individual children.

**Advocacy.** The clinical team is positioned to advocate for the systematic integration of volunteer programmes within institutional psychosocial care strategies, including securing appropriate training resources and contributing to programme evaluation [5].

## 7. Recommendations for Clinical Practice

The following six evidence-informed recommendations are proposed for paediatric oncology clinical practice and institutional policy.

**Recommendation 1: Establish mandatory pre-access training for all volunteers** *(Evidence basis: theoretical rationale and indirect empirical evidence on staff–volunteer communication and role clarity; no direct empirical evidence on volunteer training outcomes specifically).* Training should cover child development, recognition of distress and clinical deterioration, infection control, role boundaries, confidentiality, and communication with nursing staff. A structured pre-access training programme of sufficient duration to address each of these domains is recommended as a non-negotiable prerequisite for ward access, with annual refresher requirements; the specific duration should be determined by institutional context and validated against competency outcomes rather than fixed as a minimum hour threshold [5]. Where institutional resources permit, training frameworks developed by national paediatric oncology nursing bodies or hospital accreditation standards provide a useful reference point.

**Recommendation 2: Designate a volunteer coordinator within the nursing or psychosocial team** *(Evidence basis: expert consensus and psychosocial standards-of-care documents; no dedicated empirical study of coordinator models in volunteer programmes).* This role should include responsibility for scheduling, ongoing training, supervision, communication with ward nursing staff, and programme evaluation. The coordinator role should be recognised as a dedicated responsibility rather than absorbed into existing clinical workload; institutional investment in coordination infrastructure is a prerequisite for sustainable programme delivery.

**Recommendation 3: Ensure all volunteer activities are genuinely voluntary and individualised** *(Evidence basis: theoretical grounding in developmental models of autonomy, supported by indirect empirical evidence on the importance of individualisation and age-appropriate delivery).* Active, age-appropriate consent must be obtained and respected at every encounter. Activities should be adapted to the child’s developmental stage, cultural background and family values, personal interests, and clinical condition on that day [9,10,15]. In settings where family members assume extensive caregiving roles, or where attitudes towards play, stranger interaction, or professional boundaries differ from Western European norms, the model of external volunteer involvement requires cultural adaptation before implementation; nurses are best positioned to assess and communicate these contextual factors to volunteer coordinators.

**Recommendation 4: Restrict volunteer access during intensive treatment phases and periods of physical depletion** *(Evidence basis: indirect empirical evidence on cancer-related fatigue and the risks of excessive stimulation during intensive treatment).* The clinical care team must hold clear authority to suspend volunteer contact when the child’s clinical state warrants. Protocols should define explicit criteria for temporary suspension [18].

**Recommendation 5: Incorporate volunteer programme evaluation into routine quality improvement cycles** *(Evidence basis: expert consensus and psychosocial standards-of-care documents; this recommendation is not itself derived from empirical evaluation data on volunteer programmes).* Systematic data collection on child and family experience, nursing staff feedback, and volunteer outcomes should be built into programme design from inception. Validated tools such as the Paediatric Quality of Life Inventory may be adapted for this purpose. To support standardisation and audit across institutions, candidate objective indicators include: the proportion of eligible children offered volunteer contact per week; volunteer attendance and no-show rates; child- and family-reported satisfaction with volunteer contact; the number of infection-control or role-boundary incidents associated with volunteer activity; and staff-reported disruption events. A minimal shared indicator set of this kind, rather than institution-specific ad hoc evaluation, would allow programmes to be benchmarked against one another [5].

**Recommendation 6: Foster collaborative relationships between volunteers and the multidisciplinary team** *(Evidence basis: expert consensus and standards-of-care documents, supported by the single direct study of a multidisciplinary volunteer programme).* Regular joint meetings, shared documentation of volunteer activity (where appropriate), and inclusive team culture will reduce conflict, enhance effectiveness, and support the child’s experience of coherent, integrated care [5]. Collaborative structures should explicitly accommodate cultural and institutional diversity across settings; recommendations developed in Western European or North American contexts should be reviewed for local applicability before adoption, with clinical leadership playing a central role in contextual adaptation.

## 8. Discussion

Building on the evidence synthesis and risk analysis presented above, the observations below extend the review’s central finding—that volunteer-specific empirical evidence remains sparse relative to the substantial theoretical and adjacent empirical rationale for structured volunteer involvement—into implications for practice and future research.

First, the distinction between volunteer-delivered and professionally delivered psychosocial activities is rarely made explicit in the paediatric oncology literature, and this review has treated that distinction as a structural rather than incidental limitation. The indirect empirical evidence reviewed here supports the activities that volunteers are commonly asked to deliver—distraction, play, music, social engagement—but does so on the basis of professional delivery. Future research should examine volunteer-specific programmes directly, with particular attention to the effects of training intensity, programme structure, supervision quality, and volunteer–child relationship continuity on child outcomes. Standardised reporting frameworks for volunteer programmes in paediatric oncology, analogous to intervention reporting standards for clinical trials, would substantially advance the field.

Second, the heterogeneity of what constitutes volunteer involvement remains a fundamental challenge. Hospital-based volunteering encompasses diverse activities, competency profiles, and institutional structures. The operational definition proposed in this review—an unpaid individual providing structured non-medical activities in a lay capacity, outside formal educational placement—is offered as a working framework for future research and programme reporting, rather than a definitive taxonomy. Refinement through empirical study and international consensus is needed.

Third, the role of culture, family structure, and child preferences deserves greater attention than the current evidence base affords. The studies reviewed are predominantly drawn from Western European and North American settings. In contexts where family members assume extensive caregiving roles, or where attitudes towards play, stranger interaction, or professional boundaries differ, the model of external volunteer involvement may require substantial adaptation before implementation. This concern is explicitly reflected in Recommendations 3 and 6, which call for cultural assessment at the level of the individual child encounter and cultural review at the level of programme design respectively. The readership of Pediatric Blood and Cancer spans diverse clinical contexts, and the recommendations in this review should be implemented with sensitivity to local institutional and cultural conditions.

Fourth, a dimension noted in the literature that warrants acknowledgement here is the experience of healthcare professionals who volunteer in paediatric oncology settings outside their clinical role. Butterworth et al. and Strkljevic et al. both report that clinicians who volunteer describe benefits including enhanced empathy, professional growth, and renewed sense of purpose [12,13]. While tangential to this review’s primary focus on child outcomes, these findings are relevant to the clinical readership of this journal: they suggest that structured volunteer programmes may generate bidirectional value, and that clinical staff who engage in volunteer coordination or participation may themselves benefit professionally. This observation warrants dedicated investigation.

Finally, the emotional and professional impact on clinical staff—and nurses in particular, given their continuous ward presence—of coordinating volunteer programmes merits direct acknowledgement. Adding coordination responsibilities to already demanding clinical roles risks compounding staff burden—a concern that is not merely organisational but directly affects the quality and sustainability of volunteer programmes. Institutional investment in dedicated volunteer coordination infrastructure, rather than absorbing coordination into existing clinical workload, is a prerequisite for effective and sustainable programmes and is reflected in Recommendation 2.

## 9. Conclusions

Volunteers can represent a meaningful complement to professional psychosocial care for hospitalised children with cancer, supporting reductions in anxiety and pain, fostering social inclusion, and contributing to a sense of normality during a profoundly disrupting experience. Their effectiveness, however, is contingent on individualisation, appropriate training and supervision, structured coordination with the multidisciplinary clinical team, and clear institutional guidelines. Their role must be understood as explicitly complementary to—not substituting for—professional psychosocial care, and their involvement must be actively evaluated as part of routine programme quality assurance.

The multidisciplinary paediatric oncology team—with nurses occupying a particularly pivotal position given their continuous ward presence—is essential to realising the potential benefits of volunteer involvement while safeguarding children from its risks. Clinical leadership in volunteer programme design, coordination, and evaluation is not merely beneficial but essential. Rigorous prospective research, employing standardised volunteer programme definitions and validated child-reported outcome measures, is urgently needed to build the evidence base that clinical practice in this area deserves.

### Declaration of Generative AI and AI-Assisted Technologies in the Writing Process

During the preparation of this manuscript, the corresponding author used Claude (Anthropic), a generative AI tool, in four capacities. First, Claude assisted with language editing and translation of selected passages from Slovenian to English. Second, following the authors’ own independent manual search of PubMed, PsycINFO, and Google Scholar, Claude was used as a supplementary check for potentially missed relevant records; Claude was not used as a systematic tool for literature identification or selection, and all included studies were identified and selected by the authors themselves. Third, Claude drafted text for portions of the Section 2 during the manuscript’s development, including the operational definition of “volunteer,” the rationale for the narrative review design, and the evaluation of evidence in Section 4 distinguishing volunteer-delivered from professionally delivered interventions; these drafts were reviewed, substantively edited, and approved by the authors. Fourth, Claude assisted in reformatting manuscript terminology to reflect a multidisciplinary-team framing throughout the manuscript, and in generating the accompanying graphical abstract and cover letter. All AI-assisted content was reviewed, verified against primary sources where applicable, and edited by the authors to reflect their own scientific judgement, interpretation, and conclusions. The authors take full responsibility for the accuracy, integrity, and content of this publication.

## Figures and Tables

**Table 1:** Key studies informing the evidence base, highlighting the limited number of studies directly examining volunteer involvement.

Authors (Year)/Journal	Study Design	Population	*N*	Setting	Key Outcomes Relevant to Volunteer Activities	Relevance to Volunteer/Non-Medical Activities	Cited as
Ward et al. (2019) Lancet Oncol	Simulation-based epidemiological modelling	Global childhood cancer incidence data (0–14 years, 200 countries)	N/A	Global (200 countries)	Global childhood cancer incidence ~400,000/year; survival varies markedly by country income level	Contextualises the magnitude of the paediatric oncology population requiring psychosocial support	[7]
Kazak et al. (2015) Pediatr Blood Cancer	Systematic review/standards document	Children with cancer, families, paediatric oncology staff	Multi-study	Paediatric oncology centres (USA)	Evidence-based psychosocial standards of care; identifies domains of need across treatment trajectory	Framework within which volunteer activities are positioned as complementary to professional care	[2]
Phipps et al. (2005) Pediatr Blood Cancer	Prospective longitudinal observational study	Children with cancer (7–17 years) and their parents	*N* = 220	St. Jude Children’s Research Hospital, USA	Post-traumatic stress symptoms in children and parents vary by informant and time since diagnosis	Underscores sustained psychosocial burden; informs rationale for ongoing support including volunteer activities	[3]
Wiener et al. (2015) Pediatr Blood Cancer	Standards of care overview; narrative synthesis	Children with cancer, families, multidisciplinary psychosocial teams	N/A	Paediatric oncology (international)	Defines eight psychosocial standards of care; emphasises multidisciplinary approach and individualisation	Volunteer involvement positioned within psychosocial care standards; supports structured supervision	[4]
Perasso et al. (2025) Front Psychol	Multidisciplinary education programme report; narrative synthesis	Psychologists, nurses, educators and volunteers in Italian paediatric oncology (ANVOLT programme)	NR	Paediatric oncology wards, Italy (Salesi Hospital)	Play-based interventions reduce anxiety and distress; volunteer-facilitated play associated with reduced distress and high family satisfaction	Directly examines volunteer-facilitated play in paediatric oncology; most proximate reference to the review’s focus	[5]
Greenhalgh et al. (2018) Eur J Clin Invest	Methodological commentary	Not applicable (methodological paper)	N/A	Academic/methodological	Challenges hierarchy privileging systematic over narrative reviews; argues narrative reviews address distinct research questions	Methodological justification for narrative review design adopted in this manuscript	[6]
Uman et al. (2013) Cochrane Database Syst Rev	Cochrane systematic review and meta-analysis	Children and adolescents (0–18 years) undergoing needle-related procedures	13 RCTs; *N* = 1039	Various clinical settings (oncology, emergency, outpatient)	Distraction (play, music, imagery) significantly reduces procedural pain and distress; benefits maintained across age groups; moderate effect sizes	Strongest evidence base for distraction—primary mechanism through which volunteers reduce procedural pain	[8]
Nguyen et al. (2010) J Pediatr Oncol Nurs	Randomised clinical trial (RCT)	Children (7–12 years) with leukaemia undergoing lumbar puncture	*N* = 40	National Hospital of Paediatrics, Hanoi, Vietnam	Music therapy significantly reduced pain scores and physiological arousal (HR, RR) during and after lumbar puncture	Directly evidences music—a common volunteer-facilitated activity—reducing pain during a paediatric oncology procedure	[9]
Li et al. (2016) BMC Pediatr	Quasi-experimental (non-equivalent control group, pre-post design)	Hospitalised children (3–12 years; mixed diagnoses including cancer)	*N* = 304	Two acute-care public hospitals, Hong Kong	Play interventions significantly reduced negative emotions and anxiety; effect maintained across age subgroups	Provides indirect evidence for structured play reducing anxiety in hospitalised children; professional delivery	[10]
Coyne (2006) J Child Health Care	Qualitative (interviews and observations)	Hospitalised children (7–14 years) with acute illness	*N* = 11	Acute paediatric hospital wards, Ireland	Children describe loss of control and enforced dependence; peer interaction and play are highly valued; children desire normality	Children’s own perspective on hospitalisation; underpins the case for volunteer activities restoring agency and social connection	[11]
Butterworth et al. (2021) J Nurs Adm	Qualitative phenomenological study (semi-structured interviews)	Paediatric ICU nurses volunteering at a family camp for children with cancer	*N* = 12	Family oncology camp, USA	Volunteering enhanced nurses’ empathy, professional identity, and emotional resilience; renewed sense of purpose	Demonstrates bidirectional benefits: healthcare professionals who volunteer in oncology also experience professional growth	[12]
Strkljevic et al. (2024) Front Med	Scoping review	Health professionals volunteering clinical skills (all settings, international)	22 studies	International (varied)	Consistent evidence of professional growth and perspective-taking in clinicians who volunteer	Broader evidence base for involving healthcare professionals as volunteers; informs structured coordination	[13]
Hockenberry et al. (1998) J Pediatr Oncol Nurs	Integrative review	Children and adolescents with cancer (all ages)	14 studies	Paediatric oncology (international)	Cancer-related fatigue is prevalent and multidimensional; excessive stimulation worsens fatigue; individualised activity scheduling essential	Evidence base for overload risk (Section 5.1): volunteer activity must be calibrated to child’s energy and treatment phase	[14]
Bluebond-Langner et al. (2010) Nurs Clin North Am	Clinical and ethical review; qualitative evidence synthesis	Children with life-threatening or life-shortening illness and families	N/A (review)	Paediatric palliative care settings (USA/UK)	Children suppress authentic emotional expression when adults expect positivity; need for emotionally safe communication environments	Grounds risk 5.3 (emotional suppression): volunteer activities must not impose forced positivity	[15]

Abbreviations: HR = heart rate; ICU = intensive care unit; *N* = sample size; N/A = not applicable; NR = not reported; RCT = randomised controlled trial; RR = respiratory rate. Foundational theoretical monographs of Piaget [16]; Erikson [17], Lazarus & Folkman [18] and Engel [19] are not included as they are not primary empirical studies. Reference numbers correspond to the manuscript reference list.

## Data Availability

No new data were created or anylised in this study. Data sharing is not applicable.
